# Supplementary material for: Developing serum proteomics based prediction models of disease progression in ADPKD
Source: Nat Commun. 2025 Jul 19;16:6646. doi: 10.1038/s41467-025-61887-8 (PMC12274525; doi:10.1038/s41467-025-61887-8)
Supplement: Supplementary file 1 — Supplementary Information [file 41467_2025_61887_MOESM1_ESM.pdf]

Developing serum proteomics based prediction models of disease progression in ADPKD

Supplementary Figures **2**
Figure S1: Flow chart of study cohorts 2
Figure S2: Characteristics of Screening Cohort's proteome consisting of ADPKD patients 3
Figure S3: PCA of enriched GO:BP terms in ADPKD proteome 4
Figure S4: Immunoglobulin A nephropathy (IgAN) cohort – baseline characteristics and proteome. 5
Figure S5: Model accuracy across test folds 5
Figure S6: Distribution of hemoglobin beta subunit (HBB) levels across the used cohorts 6
Figure S7: Validation of the predictive models 7
Figure S8: Prediction accuracy of future eGFR 8
Figure S9: Comparison of models by using ROC curve to predict eGFR slope with a cutoff of -3 ml/min/1.73 m²/year in Screening Cohort 9
Figure S10: Principal Component Analysis (PCA) plots of the Screening Proteome 10
Figure S11: Principal Component Analysis (PCA) plots of the Validation Proteome 11

Supplementary Tables **11**
Table S1: Overview of eGFR value availability and timeframe 11
Table S2: Additional clinical characteristics of Screening and Internal/Temporal Cohorts (SC and ITC, respectively) 11
Table S3: Characteristics of patients clusters in Figure 1 12
Table S4: ADPKD cohort: Kidney function dependency of the 29 proteins selected in the integrated LIMMA/LASSO dataset of the ADPKD Screening Cohort 12
Table S5: IgAN cohort: Kidney function dependency of the 29 proteins selected in the integrated LIMMA/LASSO dataset of the ADPKD Screening Cohort 13
Table S6: Summary of the generated Proteome Model from Screening Cohort (SC) 13
Table S7: Comparison of the generated models from Screening Cohort (SC) 14
Table S8: Sample and patient sizes of three cohorts in Figure S7A-D 15
Table S9: Summary of the generated Proteome4 Model from Screening Cohort (SC) 15
Table S10: Correlation table representing the relation among proteins, eGFR and slope in Screening Cohort (SC) 15
Table S11: Comparison of the Proteome Model to Models with CST3 from Screening Cohort (SC) 16

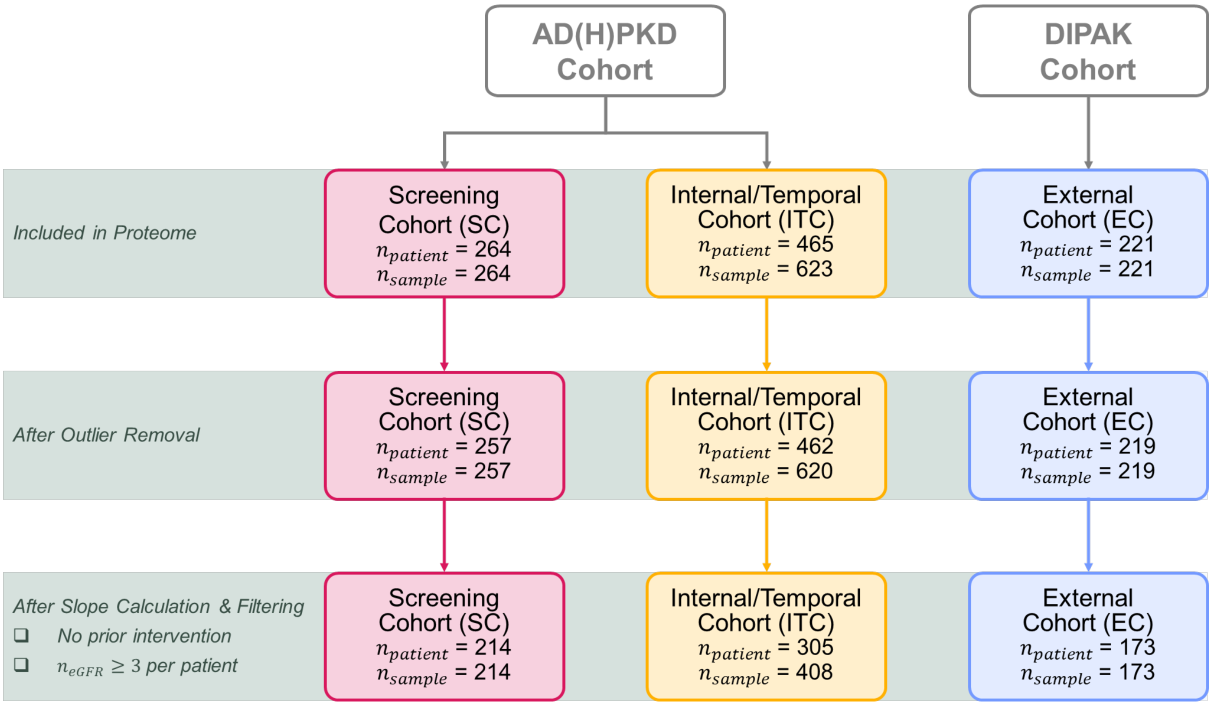

Figure S1: Flow chart of study cohorts. Blood samples derived from patients with ADPKD from the German AD(H)PKD registry contained in the Screening Cohort (SC) or Internal/Temporal Cohort (ITC) were used for proteome mass spectrometry analysis. Similarly, the External Cohort (EC) derived from the DIPAK Cohort was used. Further removal of samples and patients were performed based on quality controls, intervention, and amount of eGFR measurement. Details can be found in Methods, section eGFR Slope Calculations. The final datasets contained 214 (SC), 305 (ITC) and 173 (EC) patients respectively and were used for model generation and validation.

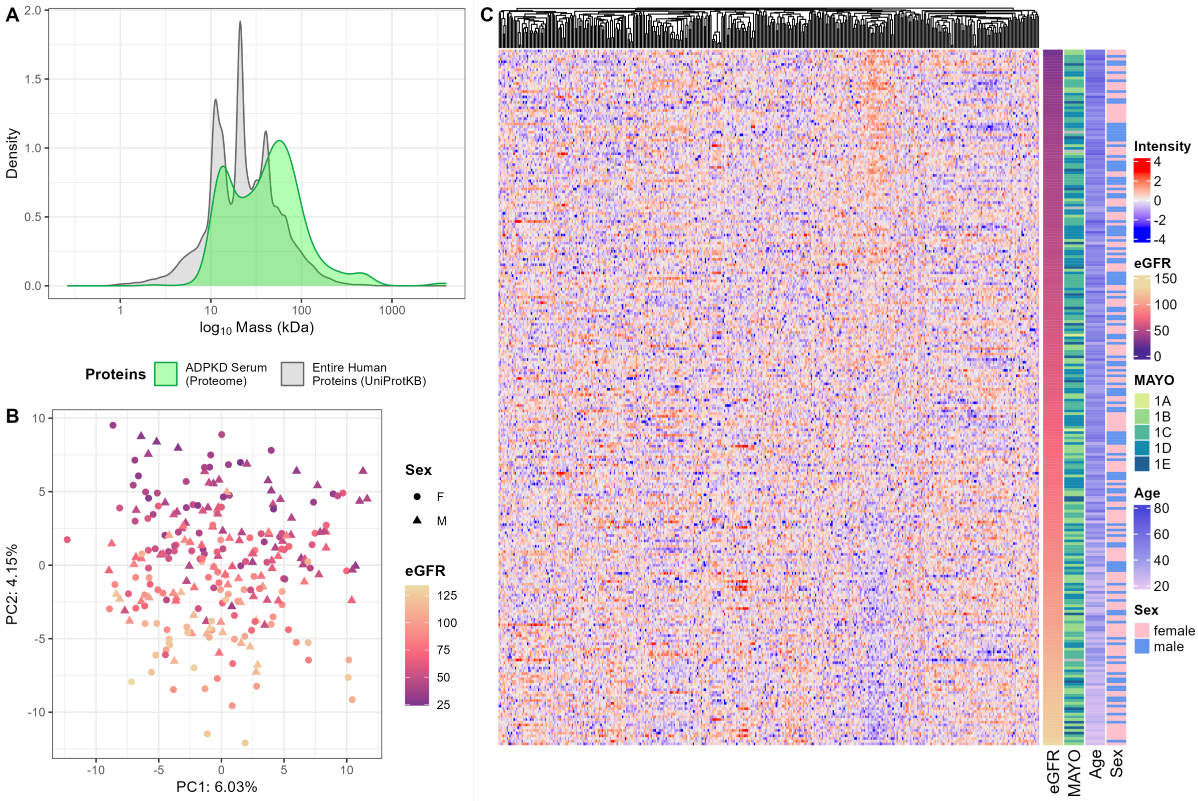

Figure S2: Characteristics of Screening Cohort's proteome consisting of ADPKD patients. A) Density plot comparing the distribution of detected proteins in patients with ADPKD (green) to the distribution of entire human proteins (gray). B) Principal Component Analysis (PCA) plot of the detected proteins in ADPKD samples. Each point represents an individual sample, and the data points are color-coded by eGFR. Females and males are represented as circles and triangles, respectively. C) Heatmap of detected proteins (n=398, in columns) and samples (n = 257, in rows), and corresponding clinical parameters. The samples were sorted according to eGFR. Source data are provided as a Source Data file.

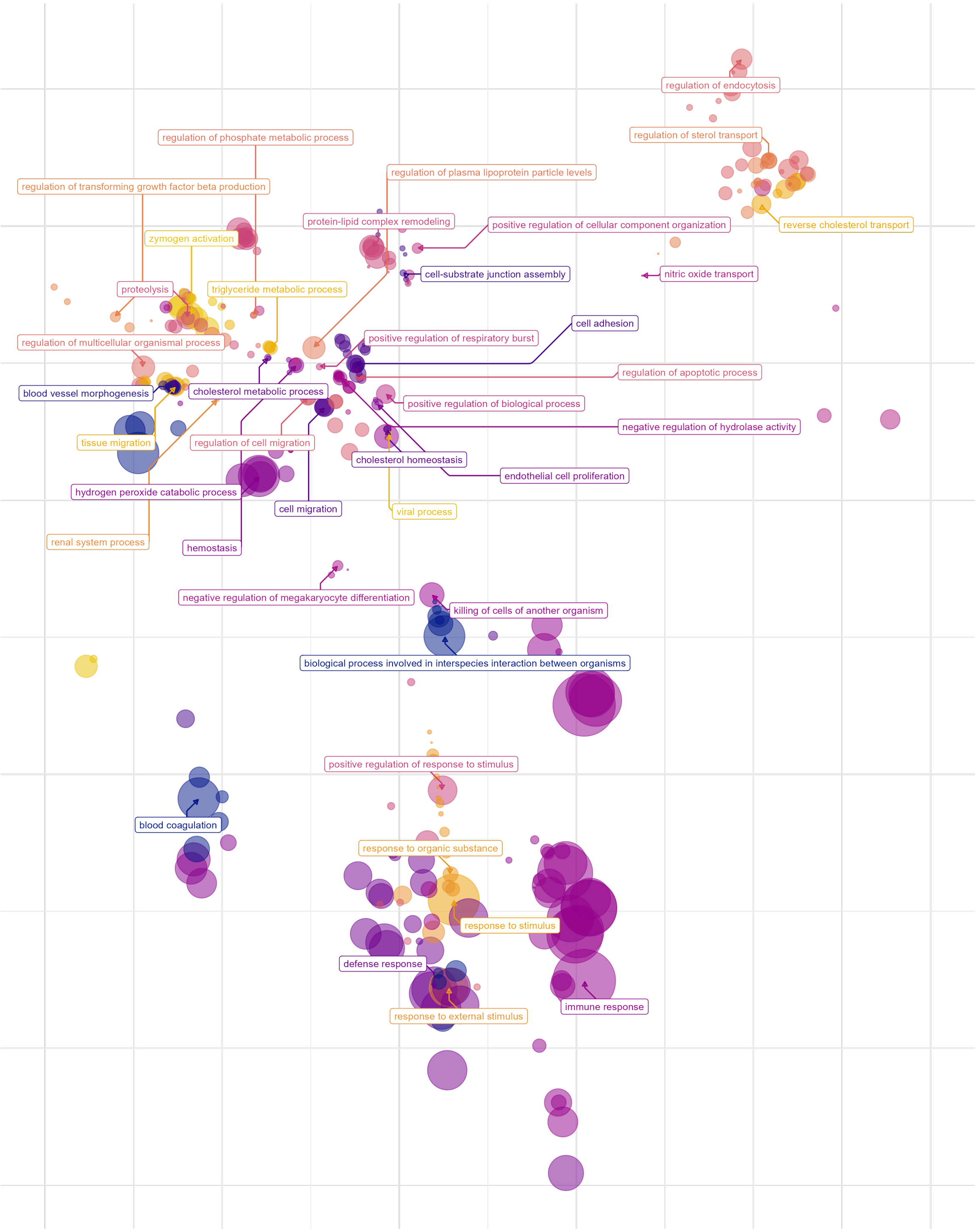

Figure S3: PCA of enriched GO:BP terms in ADPKD proteome. The generated similarity matrix of enriched GO:BP terms were subjected to PCA. The x- and y-axis are PC2 and PC1 respectively. Bubbles were color coded according to the parent terms listed in Table S4. Source data are provided as a Source Data file.

A

	IgAN Cohort
Patients, n	29
Sex (Female %)	51.7
Age (years), median (IQR)	43 (20)
eGFR (ml/min/ m^2), median (IQR)	75.6 (40.5)

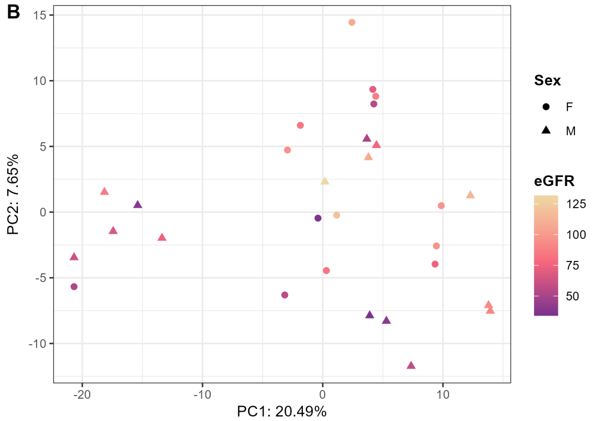

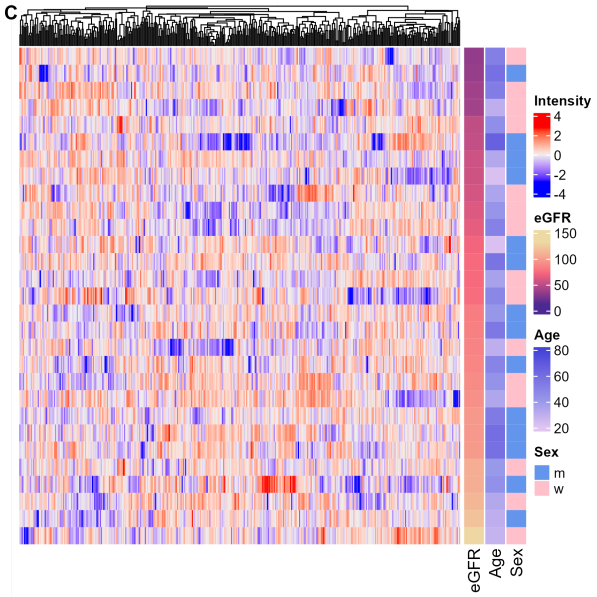

Figure S4: Immunoglobulin A nephropathy (IgAN) cohort – baseline characteristics and proteome. A) Clinical characteristics of IgAN Cohort. B) Principal Component Analysis (PCA) plot of the detected proteins in IgAN samples. Each point represents an individual sample, and the data points are color-coded by eGFR. Females and males are represented as circles and triangles, respectively. C) Heatmap of detected proteins (n=383, in columns) and samples (n = 29, in rows), and corresponding clinical parameters. The samples were sorted according to eGFR. Source data are provided as a Source Data file.

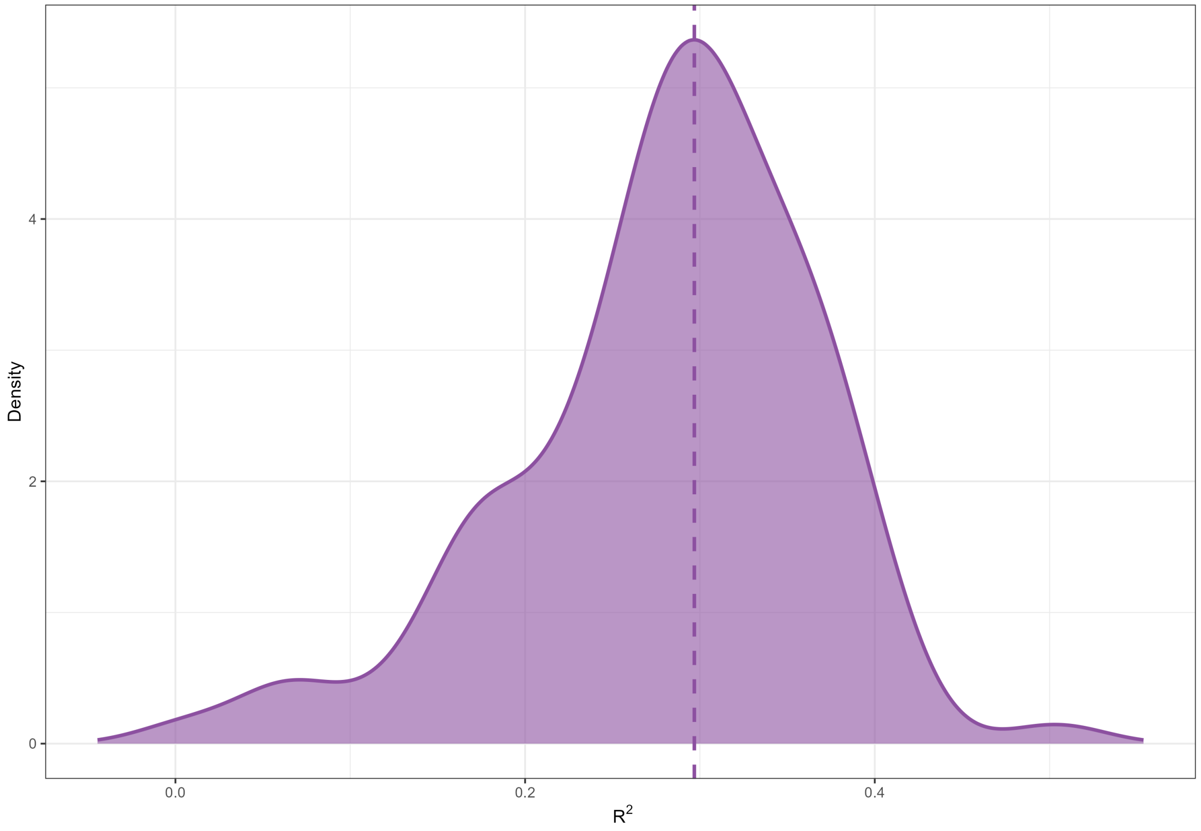

Figure S5: Model accuracy across test folds. Density plot illustrating the performance of linear regression (LR) models based on R^2 values in the test subsets of cross-validation (CV) folds. The dashed line represents the median R^2 value across the 100 tested models. Source data are provided as a Source Data file.

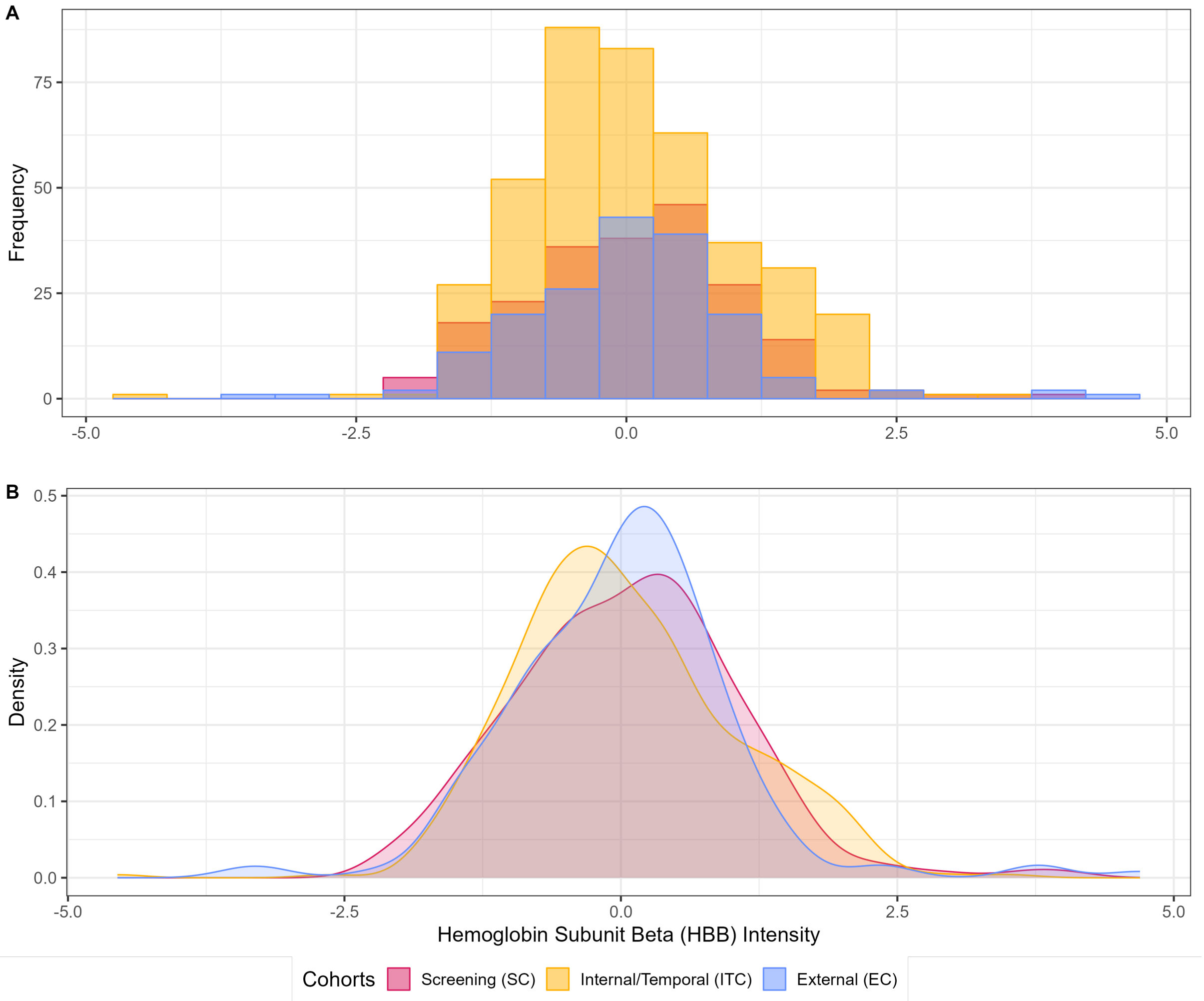

Figure S6: Distribution of hemoglobin beta subunit (HBB) levels across the used cohorts. A) Histogram depicting the frequency distribution of HBB levels within the three cohorts, highlighting the variations in distribution patterns. B) Density plot providing a smoothed representation of the HBB distribution, allowing for a clearer visualization of the distribution differences between the cohorts. Source data are provided as a Source Data file.

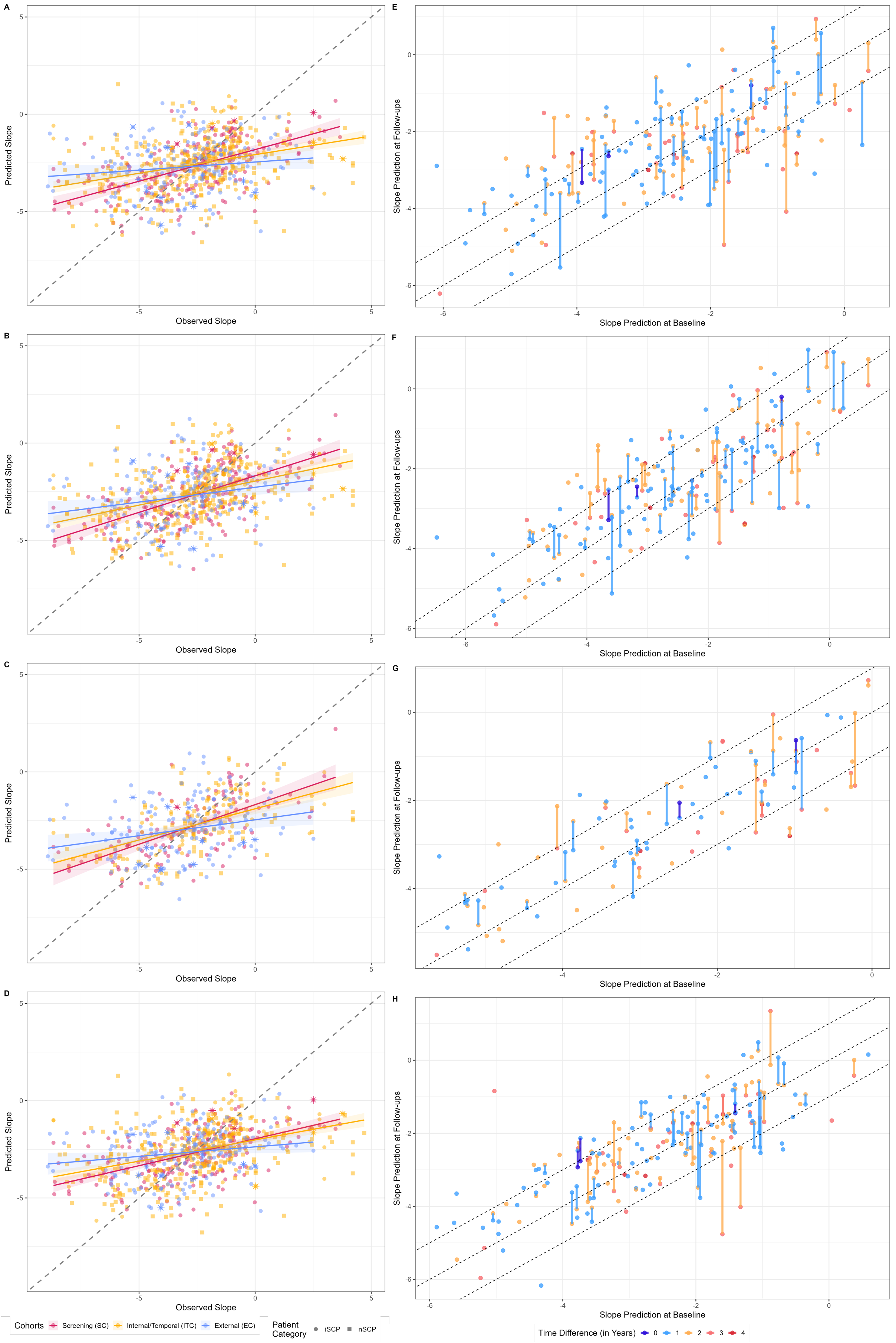
A
B
C
D
Predicted Slope
Observed Slope
E
F
G
H
Slope Prediction at Follow-ups
Slope Prediction at Baseline
Cohorts
Screening (SC)
Internal/Temporal (ITC)
External (EC)
Patient Category
iSCP
nSCP
Time Difference (in Years)
0
1
2
3
4

Figure S7: Validation of the predictive models. (A-D) Predicted slope comparison to observed slope with models, which were built in Screening Cohort (SC, red) and validated on Internal/Temporal (ITC, yellow) and External (EC, blue) Cohorts. Slope predictions made by A) Proteome, B) Combined, C) Combined Genotype and D) Proteome4 Models. The Patient Category legend indicates whether the patients included in the proteome of Screening Cohort (iSCP) but were sampled at different time points in ITC or were newly recruited (nSCP). The asterisks on the points indicate whether the samples were coming from patients who had or have been on somatostatin analogues for polycystic liver disease. The solid line is the fitted using a robust linear regression methodology and the shaded area shows bootstrapped 95% confidence interval. (E-H) Predicted slope comparison by different models for the same patients across different time points (SC and ITC). E) Proteome, F) Combined, G) Combined Genotype and H) Proteome4 Models. Each line indicates one patient and points on those lines indicate different sampling times. Color-coded for the maximum time difference between those points. Source data are provided as a Source Data file.

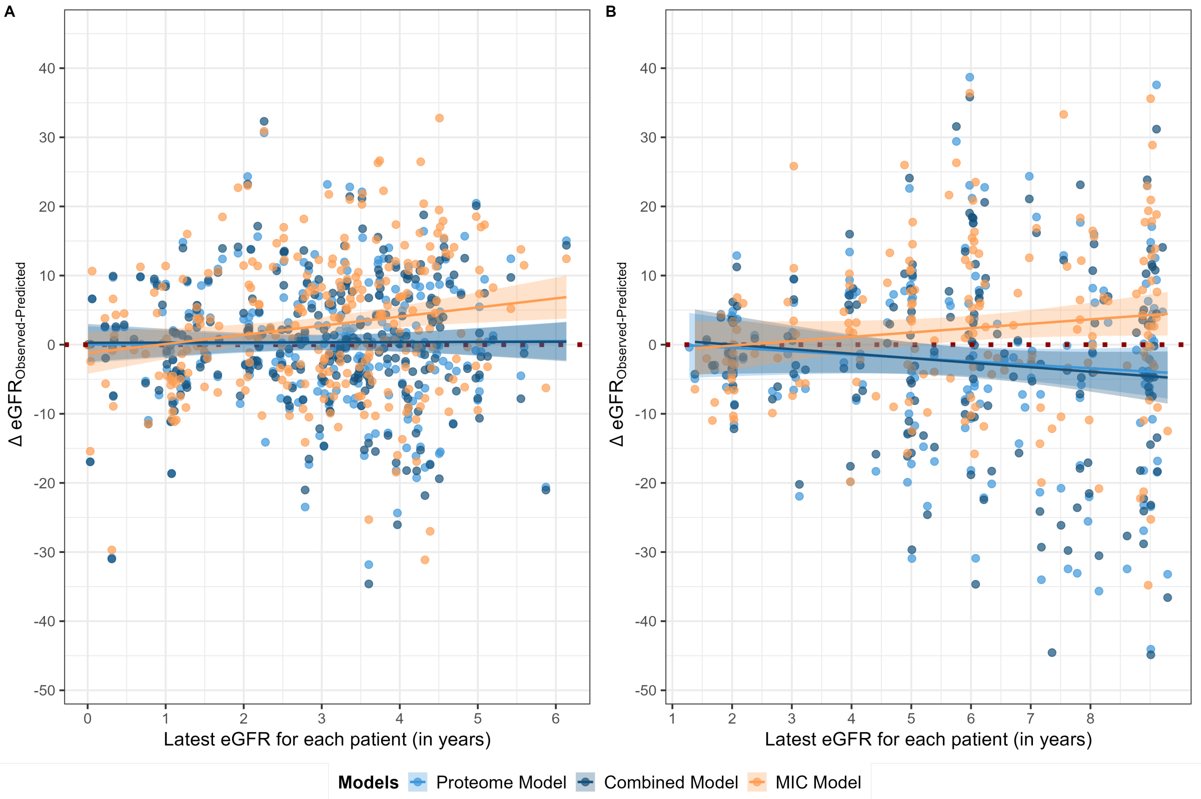

Figure S8: Prediction accuracy of future eGFR. (A) Internal/Temporal Cohort (ITC) and (B) External Cohort (EC). The last available eGFR value of each patient was predicted using the respective models. The plots show the difference between observed and predicted eGFR values (ΔeGFR = Observed - Predicted) for individual patients. Data points represent individual predictions, with models color-coded as follows: Proteome Model (n_{ITC}=278 and n_{EC}=173, blue), Combined Model (n_{ITC}=266 and n_{EC}=169, dark blue), and Mayo Imaging Classification (MIC) Model (n_{ITC}=266 and n_{EC}=169, orange). The fitted regression lines and confidence intervals illustrate the error and variability in predictions for each model. A value of ΔeGFR = 0 represents a perfect prediction, with deviations above or below indicating over- or underestimation of eGFR, respectively. The solid line is the fitted using a linear regression approach and the shaded area shows 95% confidence interval based on standard error. Source data are provided as a Source Data file.

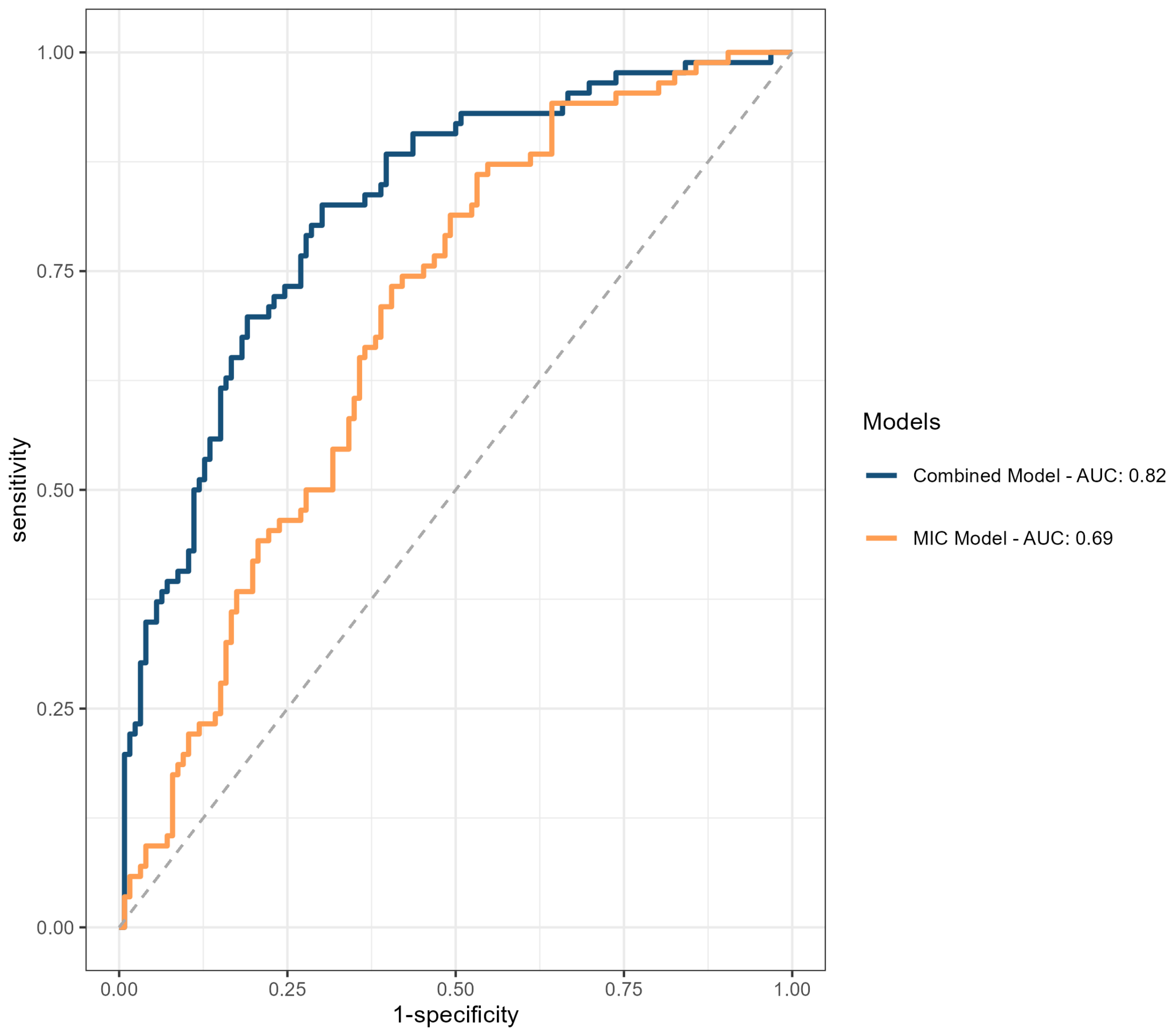

Figure S9: Comparison of models by using ROC curve to predict eGFR slope with a cutoff of -3 ml/min/1.73 m²/year in Screening Cohort. The lines are color-coded by models (n=212). Source data are provided as a Source Data file.

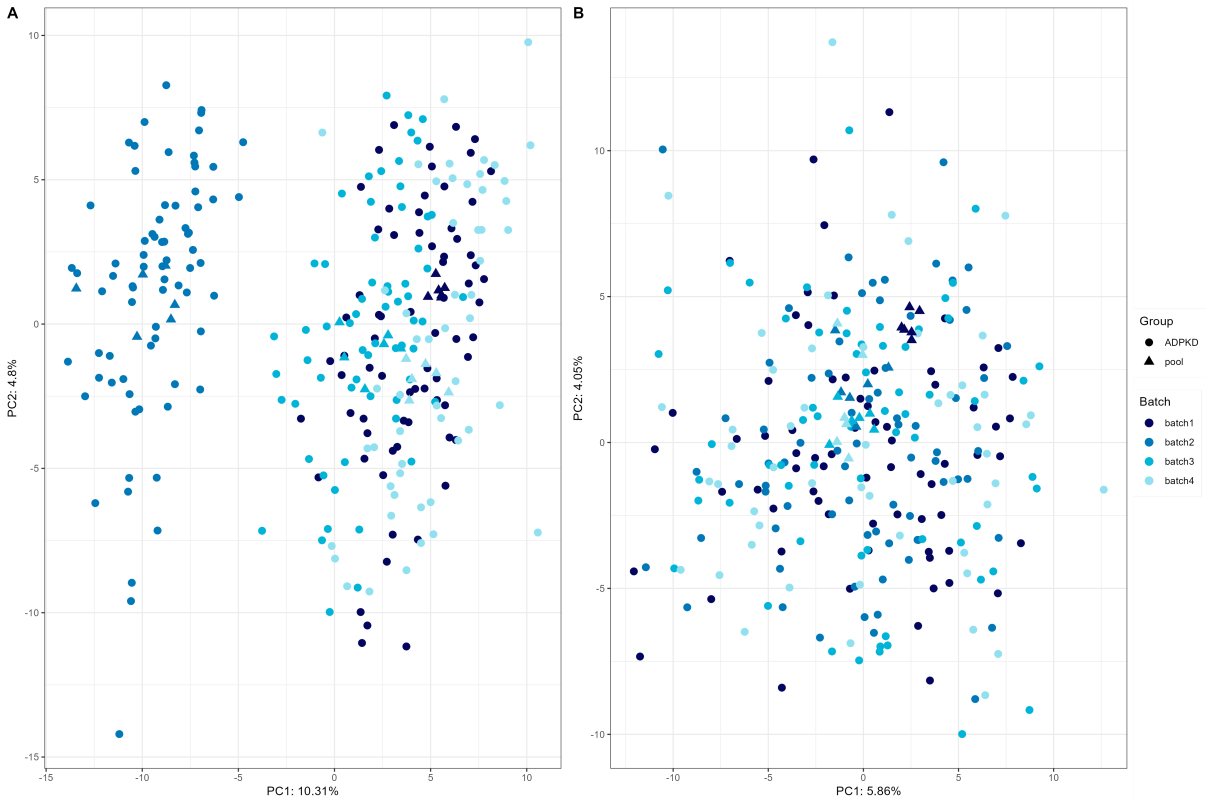

Figure S10: Principal Component Analysis (PCA) plots of the Screening Proteome. The PCA plots illustrate the variance within the proteome of Screening Cohort. A) Cohort prior to batch effect correction, exhibiting discernible clustering influenced by batch effects (n_{ADPKD}=264 and n_{pool}=24). B) Cohort post-batch effect correction, demonstrating more consistent clustering and reduced batch-related variability (n_{ADPKD}=257 and n_{pool}=24). Source data are provided as a Source Data file.

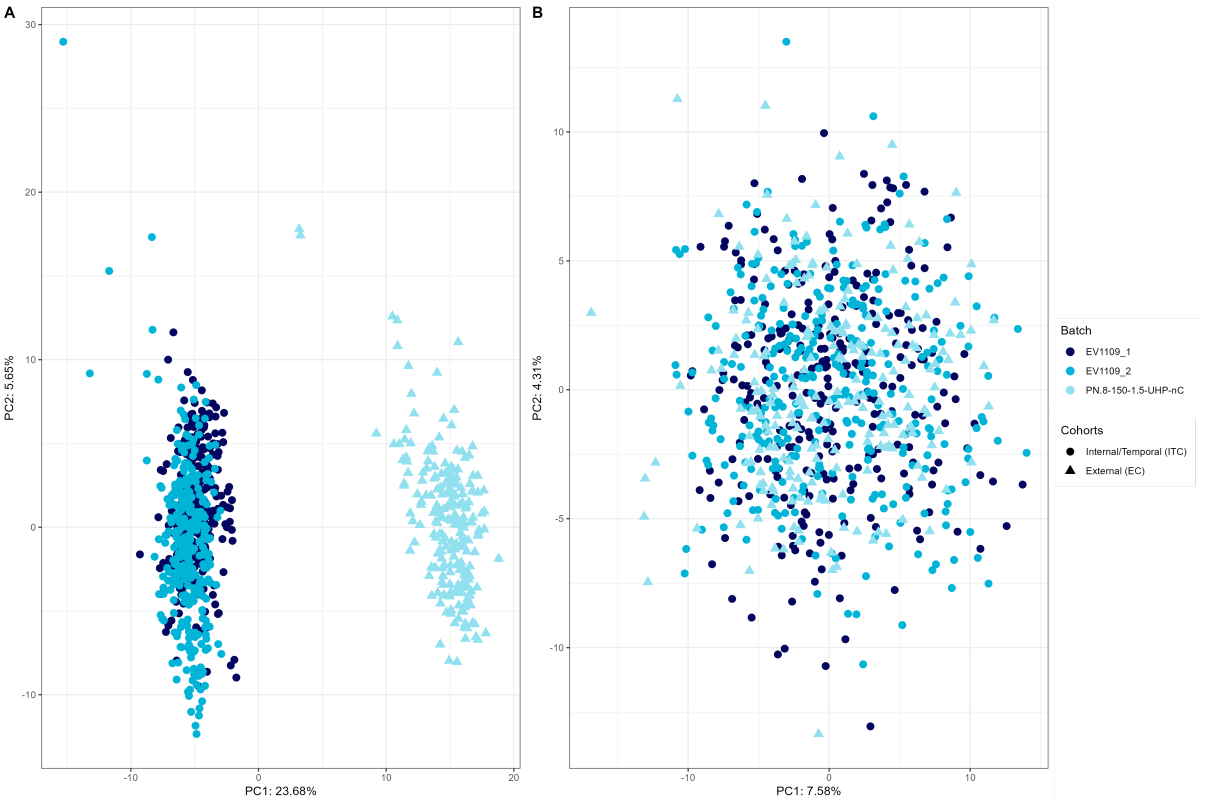

Figure S11: Principal Component Analysis (PCA) plots of the Validation Proteome. The PCA plots illustrate the variance within the proteome of Internal/Temporal and External Cohorts (ITC and EC). A) Cohort prior to batch effect correction, exhibiting discernible clustering influenced by batch effects (n_{ITC}=623 and n_{EC}=221). B) Cohort post-batch effect correction, demonstrating more consistent clustering and reduced batch-related variability (n_{ITC}=620 and n_{EC}=219). Source data are provided as a Source Data file.

Supplementary Tables

Table S1: Overview of eGFR value availability and timeframe.

	Screening Cohort (SC)	Internal/Temporal Cohort (ITC)	External Cohort (EC)
Median follow-up time, days [years]	2489 [6.8]	2329 [6.4]	2205 [6]
Mean follow-up time, days [years]	2933.8 [8]	2783.3 [7.6]	2166.8 [5.9]
Median number of eGFR measurements	10	9	7
Median time between measurements, days [years]	183 [0.5]	163 [0.4]	365 [1]

Table S2: Additional clinical characteristics of Screening and Internal/Temporal Cohorts (SC and ITC, respectively). Urological complications were defined as hematuria, flank pain, cyst infection and kidney stones. For ITC, n provided is the number of samples instead of patients since it employs several time points per patient.

	Screening Cohort (SC)	Internal/Temporal Cohort (ITC)
**Patients, n**	214	408
**Positive family history for ADPKD, n (%)**	91.6	81.6
**Arterial hypertension, n**	152	161
No hypertension	22	22
<35 years of age	66	75
≥35 years of age	61	63
Unknown age	3	1
**Urological complications, n**	214	407
No Urological complications	61	188
<35 years of age	83	121
≥35 years of age	68	96
Unknown age	2	2

Table S3: Characteristics of patients clusters in Figure 1. Comparison of clusters in terms of sex, age, eGFR and slope can be found in the last four rows. Clusters were compared to the Screening Cohort for sex distribution. IQR = Interquartile Range. Significance was denoted as stars (*<0.05, **<0.01, ***<0.001), ns = not significant. For age, eGFR and slope, two-sided t-test with Bonferroni adjustment was performed while for sex one-sided proportions test was used without adjustment due to low number of tests performed.

		Patient Cluster 1	Patient Cluster 2	Patient Cluster 3
**Patients, n**		72	69	73
**Sex**	Female	58	25	38
	Male	14	44	35
**MAYO Class**	1A-1B	36	18	10
	1C	18	26	31
	1D-1E	17	24	32
**Age (years), median (IQR)**		36.9 (21.0)	47.2 (17.5)	50.5 (10.8)
**eGFR (ml/min/1.73m^2), median (IQR)**		97.4 (28.9)	68.9 (24.3)	44.5 (16.3)
**Slope, median (IQR)**		-1.4 (2.1)	-2.5 (2.7)	-3.9 (3.1)
**Significant difference in sex between clusters to the cohort**		***	**	ns
**Significant difference among age from clusters**		2**, 3***	1**, 3***	1***, 2***
**Significant difference among eGFR from clusters**		2***, 3***	1***,3***	1***, 2***
**Significant difference among eGFR slope from clusters**		2***, 3***	1***,3***	1***, 2***

Table S4: ADPKD cohort: Kidney function dependency of the 29 proteins selected in the integrated LIMMA/LASSO dataset of the ADPKD Screening Cohort. Correlation analysis of protein abundance with eGFR at the time of biosampling. Significance is denoted as asterisks (*<0.05, **<0.01, ***<0.001). Two-sided Pearson correlation without adjustment for multiple comparisons was performed.

	Correlation with eGFR	p-value of correlation	Significance
**GPX3**	0.52	0.00	***
**SERPINF1**	-0.48	0.00	***
**AFM**	0.28	0.00	***
**CFHR1**	-0.27	0.00	***
**FERMT3**	0.19	0.01	**
**RARRES2**	-0.25	0.00	***
**CST3**	-0.65	0.00	***
**IGFBP6**	-0.63	0.00	***
**CFD**	-0.63	0.00	***
**LYZ**	-0.53	0.00	***
**ANG**	-0.34	0.00	***
**AZGP1**	-0.45	0.00	***
**AMBP**	-0.47	0.00	***
**APOA1**	0.24	0.00	***
**TF#1**	0.49	0.00	***
**RBP4**	-0.51	0.00	***
**EFEMP1**	-0.62	0.00	***
**RNASE1**	-0.50	0.00	***
**PROS1**	-0.21	0.00	**
**PCOLCE**	-0.42	0.00	***
**TF#2**	0.46	0.00	***
**SERPINF2**	0.38	0.00	***
**ORM1**	-0.23	0.00	***
**APOA4**	-0.41	0.00	***
**SERPINA6**	0.24	0.00	***
**C4BPA**	-0.26	0.00	***
**APCS**	-0.20	0.00	**
**APOA2**	0.40	0.00	***
**ORM2**	-0.13	0.06	

Table S5: IgAN cohort: Kidney function dependency of the 29 proteins selected in the integrated LIMMA/LASSO dataset of the ADPKD Screening Cohort. Correlation analysis of protein abundance with eGFR at the time of biosampling using data from the control cohort (IgAN). Significance is denoted as asterisks (*<0.05, **<0.01, ***<0.001). Two-sided Pearson correlation without adjustment for multiple comparisons was performed.

	Correlation with eGFR	p-value of correlation	Significance
**GPX3**	0.71	0.00	***
**SERPINF1**	0.13	0.51	
**AFM**	0.27	0.16	
**CFHR1**	-0.03	0.88	
**FERMT3**	0.19	0.33	
**RARRES2**	0.30	0.11	
**CST3**	-0.50	0.01	**
**IGFBP6**	-0.64	0.00	***
**CFD**	-0.62	0.00	***
**LYZ**	-0.55	0.00	**
**ANG**	-0.75	0.00	***
**AZGP1**	-0.35	0.06	
**AMBP**	-0.45	0.01	*
**APOA1**	0.14	0.46	
**TF#1**	0.13	0.49	
**RBP4**	-0.13	0.51	
**EFEMP1**	-0.25	0.19	
**RNASE1**	-0.25	0.19	
**PROS1**	-0.57	0.00	**
**PCOLCE**	0.29	0.13	
**TF#2**	-0.17	0.39	
**SERPINF2**	0.52	0.00	**
**ORM1**	-0.01	0.95	
**APOA4**	-0.36	0.05	
**SERPINA6**	-0.08	0.69	
**C4BPA**	-0.30	0.12	
**APCS**	0.05	0.79	
**APOA2**	0.05	0.78	
**ORM2**	-0.04	0.85	

Table S6: Summary of the generated Proteome Model from Screening Cohort (SC). β = Estimates, CI = Confidence interval, p = p-value, SERPINF1 = Endothelial Plasminogen Activator Inhibitor (Serpin F1), GPX3 = Glutathione Peroxidase 3, AFM = Afamin, FERMT3 = FERM Domain Containing Kindlin-3, CFHR1 = Complement Factor H Related 1, RARRES2 = Retinoic Acid Receptor Responder 2. Two-sided t-test without adjustment for multiple comparisons was performed. p-value < 0.05 is indicated in bold.

	Proteome Model		
**Predictors**	**β**	**CI**	**p**
**(Intercept)**	-2.77	-3.04 – -2.49	**<0.001**
**SERPINF1**	-0.58	-0.91 – -0.25	**0.001**
**GPX3**	0.71	0.42 – 1.00	**<0.001**
**AFM**	0.38	0.10 – 0.65	**0.008**
**FERMT3**	-0.47	-0.74 – -0.19	**0.001**
**CFHR1**	-0.35	-0.62 – -0.07	**0.014**
**RARRES2**	-0.36	-0.66 – -0.05	**0.021**
**Observations**	214		
**R^2 / R^2 adjusted**	0.318 / 0.298		

Table S7: Comparison of the generated models from Screening Cohort (SC). β = Estimates, CI = Confidence interval, p = p-value, SERPINF1 = Endothelial Plasminogen Activator Inhibitor (Serpin F1), GPX3 = Glutathione Peroxidase 3, AFM = Afamin, FERMT3 = FERM Domain Containing Kindlin-3, CFHR1 = Complement Factor H Related 1, RARRES2 = Retinoic Acid Receptor Responder 2, Age in years, eGFR in ml/min/1.73m^2, MAYO [Mid] = MAYO Class 1C, MAYO [More] = MAYO Class 1D-1E, PKD1 [NT] = PKD1 non-truncating mutations, PKD1 [T] = PKD1 truncating mutations. Two-sided t-test without adjustment for multiple comparisons was performed. p-value < 0.05 is indicated in bold.

	Proteome Model			Clinical Model			Clinical Genotype Model			Combined Model			Combined Genotype Model		
**Predictors**	**β**	**CI**	**p**	**β**	**CI**	**p**	**β**	**CI**	**p**	**β**	**CI**	**p**	**β**	**CI**	**p**
**(Intercept)**	-2.71	-3.10 – -2.31	**<0.001**	-7.76	-12.31 – -3.20	**0.001**	-3.72	-8.87 – 1.43	0.155	-4.78	-9.75 – 0.19	0.059	-2.38	-7.91 – 3.15	0.395
**SERPINF1**	-0.53	-1.01 – -0.05	**0.029**							-0.22	-0.75 – 0.32	0.427	-0.22	-0.75 – 0.31	0.418
**GPX3**	0.69	0.31 – 1.08	**0.001**							0.42	-0.04 – 0.89	0.075	0.41	-0.06 – 0.88	0.090
**AFM**	0.46	0.07 – 0.84	**0.020**							0.28	-0.13 – 0.69	0.180	0.24	-0.17 – 0.65	0.255
**FERMT3**	-0.52	-0.90 – -0.14	**0.008**							-0.48	-0.87 – -0.09	**0.016**	-0.39	-0.78 – 0.01	0.055
**CFHR1**	-0.35	-0.75 – 0.05	0.087							-0.30	-0.72 – 0.11	0.152	-0.23	-0.65 – 0.19	0.276
**RARRES2**	-0.54	-0.98 – -0.11	**0.014**							-0.46	-0.90 – -0.02	**0.041**	-0.38	-0.82 – 0.07	0.095
**Age**				0.04	-0.02 – 0.10	0.145	0.00	-0.06 – 0.06	0.995	0.02	-0.04 – 0.08	0.584	-0.01	-0.08 – 0.06	0.778
**Sex [Male]**				-0.42	-1.26 – 0.42	0.323	-0.53	-1.34 – 0.29	0.203	-0.46	-1.31 – 0.38	0.280	-0.52	-1.36 – 0.32	0.222
**eGFR**				0.05	0.03 – 0.08	**<0.001**	0.04	0.01 – 0.06	**0.003**	0.03	-0.01 – 0.06	0.113	0.02	-0.01 – 0.05	0.248
**MAYO [1C]**				-0.48	-1.59 – 0.63	0.391	-0.46	-1.54 – 0.62	0.399	-0.19	-1.30 – 0.92	0.734	-0.20	-1.31 – 0.90	0.716
**MAYO [1D-1E]**				-0.82	-2.12 – 0.47	0.210	-1.02	-2.30 – 0.25	0.113	-0.54	-1.84 – 0.77	0.415	-0.69	-2.00 – 0.62	0.296
**PKD1 [NT]**							-1.70	-2.97 – -0.43	**0.009**				-1.11	-2.42 – 0.20	0.095
**PKD1 [T]**							-1.41	-2.44 – -0.39	**0.007**				-0.97	-2.03 – 0.09	0.074
**Observations**	114			114			114			114			114		
**R^2 / R^2 adjusted**	0.342 / 0.305			0.288 / 0.255			0.346 / 0.303			0.385 / 0.319			0.408 / 0.331		

Table S8: Sample and patient sizes of three cohorts in Figure S7A-C. SC = Screening Cohort, ITC = Internal/Temporal Cohort, EC = External Cohort.

		Proteome Model	Combined Model	Combined Genotype Model
SC	Sample Size	214	212	114
	Patient Size	214	212	114
ITC	Sample Size	408	392	169
	Patient Size	305	291	114
EC	Sample Size	173	169	158
	Patient Size	173	169	158

Table S9: Summary of the generated Proteome4 Model from Screening Cohort (SC). β = Estimates, CI = Confidence interval, p = p-value, SERPINF1 = Endothelial Plasminogen Activator Inhibitor (Serpin F1), GPX3 = Glutathione Peroxidase 3, AFM = Afamin, CFHR1 = Complement Factor H Related 1. Two-sided t-test without adjustment for multiple comparisons was performed. p-value < 0.05 is indicated in bold.

	Proteome4 Model		
**Predictors**	**β**	**CI**	**p**
**(Intercept)**	-2.78	-3.06 – -2.49	**<0.001**
**SERPINF1**	-0.68	-1.00 – -0.37	**<0.001**
**GPX3**	0.68	0.38 – 0.98	**<0.001**
**AFM**	0.39	0.11 – 0.68	**0.007**
**CFHR1**	-0.31	-0.59 – -0.02	**0.033**
**Observations**	214		
**R^2 / R^2 adjusted**	0.265 / 0.251		

Table S10: Correlation table representing the relation among proteins, eGFR and slope in Screening Cohort (SC). SERPINF1 = Endothelial Plasminogen Activator Inhibitor (Serpin F1), GPX3 = Glutathione Peroxidase 3, AFM = Afamin, FERMT3 = FERM Domain Containing Kindlin-3, CFHR1 = Complement Factor H Related 1, RARRES2 = Retinoic Acid Receptor Responder 2, CST3 = Cystatin-C, eGFR = estimated Glomerular Filtration Rate, Slope = annual eGFR decline. Two-sided Pearson correlation without adjustment for multiple comparisons was performed.

	GPX3	SERPINF1	AFM	CFHR1	FERMT3	RARRES2	CST3	eGFR	Slope
**GPX3**	1.00	-0.30	0.08	-0.17	0.14	-0.15	-0.39	0.52	0.40
**SERPINF1**	-0.30	1.00	0.12	0.19	-0.12	0.38	0.40	-0.48	-0.36
**AFM**	0.08	0.12	1.00	-0.03	0.00	0.00	-0.19	0.28	0.16
**CFHR1**	-0.17	0.19	-0.03	1.00	-0.02	-0.05	0.30	-0.27	-0.23
**FERMT3**	0.14	-0.12	0.00	-0.02	1.00	-0.09	-0.05	0.19	-0.11
**RARRES2**	-0.15	0.38	0.00	-0.05	-0.09	1.00	0.13	-0.25	-0.25
**CST3**	-0.39	0.40	-0.19	0.30	-0.05	0.13	1.00	-0.65	-0.38
**eGFR**	0.52	-0.48	0.28	-0.27	0.19	-0.25	-0.65	1.00	0.40
**Slope**	0.40	-0.36	0.16	-0.23	-0.11	-0.25	-0.38	0.40	1.00

Table S11: Comparison of the Proteome Model to Models with CST3 from Screening Cohort (SC). β = Estimates, CI = Confidence interval, p = p-value, SERPINF1 = Endothelial Plasminogen Activator Inhibitor (Serpin F1), GPX3 = Glutathione Peroxidase 3, AFM = Afamin, FERMT3 = FERM Domain Containing Kindlin-3, CFHR1 = Complement Factor H Related 1, RARRES2 = Retinoic Acid Receptor Responder 2, CST3 = Cystatin C. Two-sided t-test without adjustment for multiple comparisons was performed. p-value < 0.05 is indicated in bold.

	**Proteome Model**			**Model 1**			**Model 2**			**Model 3**			**Model 4**		
**Predictors**	**β**	**CI**	**p**	**β**	**CI**	**p**	**β**	**CI**	**p**	**β**	**CI**	**p**	**β**	**CI**	**p**
**(Intercept)**	-2.77	-3.04 – -2.49	**<0.001**	-2.76	-3.04 – -2.49	**<0.001**	-2.76	-3.04 – -2.49	**<0.001**	-2.72	-3.00 – -2.44	**<0.001**	-2.76	-3.04 – -2.49	**<0.001**
**SERPINF1**	-0.58	-0.91 – -0.25	**0.001**	-0.52	-0.86 – -0.17	**0.004**				-0.58	-0.94 – -0.23	**0.001**	-0.48	-0.83 – -0.13	**0.007**
**GPX3**	0.71	0.42 – 1.00	**<0.001**	0.65	0.35 – 0.96	**<0.001**	0.69	0.39 – 1.00	**<0.001**				0.64	0.33 – 0.94	**<0.001**
**AFM**	0.38	0.10 – 0.65	**0.008**	0.31	0.03 – 0.60	**0.031**	0.23	-0.05 – 0.51	0.106	0.34	0.05 – 0.64	**0.022**	0.32	0.03 – 0.60	**0.028**
**FERMT3**	-0.47	-0.74 – -0.19	**0.001**	-0.46	-0.73 – -0.18	**0.001**	-0.43	-0.71 – -0.15	**0.003**	-0.40	-0.68 – -0.11	**0.006**	-0.46	-0.73 – -0.19	**0.001**
**CFHR1**	-0.35	-0.62 – -0.07	**0.014**				-0.33	-0.61 – -0.04	**0.024**	-0.32	-0.61 – -0.03	**0.033**	-0.29	-0.57 – -0.01	**0.046**
**RARRES2**	-0.36	-0.66 – -0.05	**0.021**	-0.32	-0.62 – -0.02	**0.038**	-0.51	-0.80 – -0.23	**0.001**	-0.39	-0.70 – -0.07	**0.015**	-0.36	-0.66 – -0.06	**0.019**
**CST3**				-0.38	-0.70 – -0.06	**0.021**	-0.45	-0.77 – -0.13	**0.006**	-0.49	-0.82 – -0.16	**0.003**	-0.31	-0.64 – 0.02	0.068
**Observations**	214			214			214			214			214		
**R^2 / R^2 adjusted**	0.318 / 0.298			0.315 / 0.296			0.304 / 0.284			0.273 / 0.251			0.329 / 0.306		
