# Supplementary material for: Developing serum proteomics based prediction models of disease progression in ADPKD
Source: Nat Commun. 2025 Jul 19;16:6646. doi: 10.1038/s41467-025-61887-8 (PMC12274525; doi:10.1038/s41467-025-61887-8)
Supplement: Supplementary file 2 — Description of Additional Supplementary Files [file 41467_2025_61887_MOESM2_ESM.pdf]

Description of Additional Supplementary Files

Supplementary Data S1: Summary of the enriched terms in ADPKD proteome. One-sided Fisher's test with FDR adjustment was performed.

Supplementary Data S2: Summary of the parent enriched GO:BP terms from Table S3.

Supplementary Data S3: Summary of the enriched terms of selected features (LIMMA and LASSO sets). One-sided Fisher's test with FDR adjustment was performed.

Supplementary Data S4: Summary of the enriched terms in the protein clusters described in Figure 1. One-sided Fisher's test with FDR adjustment was performed.

Supplementary Data S5: Summary of the parent enriched GO:BP terms from Table S6.
