# Supplementary material for: Developing serum proteomics based prediction models of disease progression in ADPKD
Source: Nat Commun. 2025 Jul 19;16:6646. doi: 10.1038/s41467-025-61887-8 (PMC12274525; doi:10.1038/s41467-025-61887-8)
Supplement: Supplementary file 8 — Reporting Summary [file 41467_2025_61887_MOESM8_ESM.pdf]

nature portfolio

Corresponding author(s): Roman-Ulrich-Müller & NCOMMS-24-73470

Last updated by author(s): Jun 4, 2025

Reporting Summary

Nature Portfolio wishes to improve the reproducibility of the work that we publish. This form provides structure for consistency and transparency in reporting. For further information on Nature Portfolio policies, see our Editorial Policies and the Editorial Policy Checklist.

Statistics

For all statistical analyses, confirm that the following items are present in the figure legend, table legend, main text, or Methods section.

n/a	Confirmed	
☐	☒	The exact sample size (*n*) for each experimental group/condition, given as a discrete number and unit of measurement
☐	☒	A statement on whether measurements were taken from distinct samples or whether the same sample was measured repeatedly
☐	☒	The statistical test(s) used AND whether they are one- or two-sided<br>*Only common tests should be described solely by name; describe more complex techniques in the Methods section.*
☐	☒	A description of all covariates tested
☐	☒	A description of any assumptions or corrections, such as tests of normality and adjustment for multiple comparisons
☐	☒	A full description of the statistical parameters including central tendency (e.g. means) or other basic estimates (e.g. regression coefficient) AND variation (e.g. standard deviation) or associated estimates of uncertainty (e.g. confidence intervals)
☐	☒	For null hypothesis testing, the test statistic (e.g. *F*, *t*, *r*) with confidence intervals, effect sizes, degrees of freedom and *P* value noted<br>*Give P values as exact values whenever suitable.*
☒	☐	For Bayesian analysis, information on the choice of priors and Markov chain Monte Carlo settings
☒	☐	For hierarchical and complex designs, identification of the appropriate level for tests and full reporting of outcomes
☒	☐	Estimates of effect sizes (e.g. Cohen's *d*, Pearson's *r*), indicating how they were calculated

Our web collection on statistics for biologists contains articles on many of the points above.

Software and code

Policy information about availability of computer code

Data collection: Custom code was used and will be provided upon publication via GitHub https://github.com/handeaydogan/slope-models-adpkd

Data analysis:

```
Session info
R version 4.3.2 (2023-10-31 ucrt)
Platform: x86_64-w64-mingw32/x64 (64-bit)
Running under: Windows 11 x64 (build 22000)

Matrix products: default


locale:
[1] LC_COLLATE=English_Germany.utf8  LC_CTYPE=English_Germany.utf8    LC_MONETARY=English_Germany.utf8
[4] LC_NUMERIC=C                     LC_TIME=English_Germany.utf8

time zone: Europe/Berlin
tzcode source: internal

attached base packages:
[1] grid      stats     graphics  grDevices utils     datasets  methods   base
```

```
other packages:
 [1] "abind_1.4-5"          "affy_1.80.0"          "affyio_1.72.0"        "AFR_0.3.5"            "annotate_1.80.0"
 [6] "AnnotationDbi_1.64.1" "askpass_1.2.0"        "attached):"           "backports_1.4.1"      "base64enc_0.1-3"
[11] "bayestestR_0.13.1"    "Biobase_2.62.0"       "BiocGenerics_0.48.1"  "BiocManager_1.30.22"  "BiocParallel_1.36.0"
[16] "Biostrings_2.70.1"    "bit_4.0.5"            "bit64_4.0.5"          "bitops_1.0-7"         "blob_1.2.4"
[21] "boot_1.3-28.1"        "broom_1.0.5"          "cachem_1.0.8"         "Cairo_1.6-2"          "car_3.1-2"
[26] "carData_3.0-5"        "caret_6.0-94"         "cellranger_1.1.0"     "checkmate_2.3.1"      "circlize_0.4.15"
[31] "class_7.3-22"         "cli_3.6.2"            "clue_0.3-65"          "cluster_2.1.4"        "coda_0.19-4"
[36] "codetools_0.2-19"     "colorspace_2.1-0"     "compiler_4.3.2"       "ComplexHeatmap_2.18.0" "cowplot_1.1.2"
[41] "crayon_1.5.2"         "curl_5.2.0"           "data.table_1.14.10"   "datawizard_0.9.1"     "DBI_1.2.1"
[46] "diann_1.0.1"          "digest_0.6.34"        "doParallel_1.0.17"    "dplyr_1.1.4"          "edgeR_4.0.11"
[51] "effectsize_0.8.6"     "ellipsis_0.3.2"       "emmeans_1.9.0"        "estimability_1.4.1"   "evaluate_0.23"
[56] "fansi_1.0.6"          "farver_2.1.1"         "fastmap_1.1.1"        "forcats_1.0.0"        "foreach_1.5.2"
[61] "forecast_8.21.1"      "foreign_0.8-85"       "formatR_1.14"         "Formula_1.2-5"        "fracdiff_1.5-3"
[66] "fs_1.6.3"             "futile.logger_1.4.3"  "futile.options_1.0.1" "future.apply_1.11.1"  "future_1.33.1"
[71] "genefilter_1.84.0"    "generics_0.1.3"       "GenomeInfoDb_1.38.5"  "GenomeInfoDbData_1.2.11" "GetoptLong_1.0.5"
[76] "ggeffects_1.3.4"      "ggplot2_3.4.4"        "ggplot2_3.5.1"        "ggpubr_0.6.0"         "ggrepel_0.9.5"
[81] "ggsignif_0.6.4"       "glmnet_4.1-8"         "GlobalOptions_0.1.2"  "globals_0.16.2"       "glue_1.7.0"
[86] "GO.db_3.18.0"         "goftest_1.2-3"        "GOSemSim_2.28.1"      "gower_1.0.1"          "gprofiler2_0.2.2"
[91] "grid_4.3.2"           "gridBase_0.4-7"       "gridExtra_2.3"        "gtable_0.3.4"         "hardhat_1.3.0"
[96] "haven_2.5.4"          "Hmisc_5.1-1"          "hms_1.1.3"            "hopach_2.62.0"        "htmlTable_2.4.2"
[101] "htmltools_0.5.7"     "htmlwidgets_1.6.4"    "httpuv_1.6.13"        "httr_1.4.7"           "igraph_2.0.1.1"
[106] "insight_0.19.7"      "ipred_0.9-14"         "IRanges_2.36.0"       "iterators_1.0.14"     "janitor_2.2.0"
[111] "jsonlite_1.8.8"      "KEGGREST_1.42.0"      "knitr_1.45"           "labeling_0.4.3"       "labelled_2.12.0"
[116] "lambda.r_1.2.4"      "later_1.3.2"          "lattice_0.21-9"       "lava_1.7.3"           "lazyeval_0.2.2"
[121] "lifecycle_1.0.4"     "limma_3.58.1"         "listenv_0.9.0"        "lme4_1.1-35.1"        "lmtest_0.9-40"
[126] "loaded"              "locfit_1.5-9.8"       "lubridate_1.9.3"      "magick_2.8.6"         "magrittr_2.0.3"
[131] "makeunique_1.0.0"    "MASS_7.3-60"          "Matrix_1.6-1.1"       "MatrixGenerics_1.14.0" "matrixStats_1.2.0"
[136] "memoise_2.0.1"       "mgcv_1.9-0"           "mime_0.12"            "minqa_1.2.6"          "ModelMetrics_1.2.2.2"
[141] "modelr_0.1.11"       "multcomp_1.4-25"      "munsell_0.5.0"        "mvtnorm_1.2-4"        "namespace"
[146] "nlme_3.1-163"        "nloptr_2.0.3"         "NLP_0.2-1"            "nnet_7.3-19"          "nortest_1.0-4"
[151] "olsrr_0.6.0"         "openssl_2.1.1"        "openxlsx_4.2.5.2"     "org.Hs.eg.db_3.18.0"  "parallel_4.3.2"
[156] "parallelly_1.36.0"   "parameters_0.21.3"    "performance_0.10.8"   "pheatmap_1.0.12"      "pillar_1.9.0"
[161] "pkgconfig_2.0.3"     "plotly_4.10.4"        "plyr_1.8.9"           "png_0.1-8"            "preprocessCore_1.64.0"
[166] "pROC_1.18.5"         "prodlim_2023.08.28"   "promises_1.2.1"       "purrr_1.0.2"          "quadprog_1.5-8"
[171] "quantmod_0.4.25"     "R6_2.5.1"             "ragg_1.2.7"           "randomForest_4.7-1.1" "rcartocolor_2.1.1"
[176] "RColorBrewer_1.1-3"  "Rcpp_1.0.12"          "RcppEigen_0.3.3.9.4"  "RCurl_1.98-1.14"      "readr_2.1.5"
[181] "readxl_1.4.3"        "recipes_1.0.9"        "reshape2_1.4.4"       "reticulate_1.34.0"    "rJava_1.0-11"
[186] "rjson_0.2.21"        "rjson_0.2.23"         "rlang_1.1.3"          "rmarkdown_2.25"       "rpart_4.1.21"
[191] "rrvgo_1.14.1"        "RSpectra_0.16-1"      "RSQLite_2.3.4"        "rstatix_0.7.2"        "rstudioapi_0.15.0"
[196] "S4Vectors_0.40.2"    "sandwich_3.1-0"       "scales_1.3.0"         "shape_1.4.6"          "shiny_1.8.0"
[201] "sjlabelled_1.2.0"    "sjmisc_2.8.9"         "sjPlot_2.8.15"        "sjstats_0.18.2"       "slam_0.1-50"
[206] "snakecase_0.11.1"    "splines_4.3.2"        "statmod_1.5.0"        "stats4_4.3.2"         "stringi_1.8.3"
[211] "stringr_1.5.1"       "survival_3.5-7"       "sva_3.50.0"           "systemfonts_1.0.5"    "textshaping_0.3.7"
[216] "TH.data_1.1-2"       "tibble_3.2.1"         "tidyr_1.3.0"          "tidyselect_1.2.0"     "tidyverse_2.0.0"
[221] "timechange_0.3.0"    "timeDate_4032.109"    "tm_0.7-11"            "tools_4.3.2"          "treemap_2.4-4"
[226] "tseries_0.10-55"     "TTR_0.24.4"           "tzdb_0.4.0"           "umap_0.2.10.0"        "urca_1.3-3"
[231] "utf8_1.2.4"          "vctrs_0.6.5"          "VennDiagram_1.7.3"    "viridisLite_0.4.2"    "vroom_1.6.5"
[236] "vsn_3.70.0"          "withr_3.0.0"          "wordcloud_2.6"        "xfun_0.41"            "xfun_0.51"
[241] "xlsx_0.6.5"          "xlsxjars_0.6.1"       "XML_3.99-0.16"        "xml2_1.3.6"           "xtable_1.8-4"
[246] "xts_0.13.2"          "XVector_0.42.0"       "yulab.utils_0.1.4"    "yulab.utils_0.2.0"    "zeroSum_2.0.6"
[251] "zip_2.3.0"           "zlibbioc_1.48.0"      "zoo_1.8-12"
```

For manuscripts utilizing custom algorithms or software that are central to the research but not yet described in published literature, software must be made available to editors and reviewers. We strongly encourage code deposition in a community repository (e.g. GitHub). See the Nature Portfolio guidelines for submitting code & software for further information.

Data

Policy information about availability of data

All manuscripts must include a data availability statement. This statement should provide the following information, where applicable:

- Accession codes, unique identifiers, or web links for publicly available datasets
- A description of any restrictions on data availability
- For clinical datasets or third party data, please ensure that the statement adheres to our policy

The data underlying individual figures are provided in the source data file in an anonymized fashion. The full proteomics and clinical data supporting the findings of this study are not publicly archived to ensure data protection of study participants and minimize the risk of re-identification. Access to the dataset may be granted upon direct request to the corresponding authors depending on the nature of research questions aligning with the aims of the study to which participants provided consent and the ability to ensure data protection. To ensure this goal, data sharing will depend on a signed bilateral data transfer agreement.

Interested researchers should contact the corresponding authors at roman-ulrich.mueller@uk-koeln.de or philipp.antczak@uk-koeln.de, including a description of the intended use and any institutional affiliations. The corresponding author will respond within 10 business days of receiving a complete request. Access will be provided only to individuals or institutions that meet the conditions outlined above.
The data will remain available for a minimum of ten years following publication, unless otherwise restricted by law or institutional policy.

Research involving human participants, their data, or biological material

Policy information about studies with human participants or human data. See also policy information about sex, gender (identity/presentation), and sexual orientation and race, ethnicity and racism.

Reporting on sex and gender	Term sex was used as a biological attribute. Sex was considered in the study design and findings apply to both sex. It was determined based on assigned at birth. Consent was obtained.
Reporting on race, ethnicity, or other socially relevant groupings	All participants were of self-reported Caucasian background; no additional socially relevant groupings were applicable in this study.
Population characteristics	Age, hypertension, available genotype information and urological complications in the manuscript and supplementary
Recruitment	The Screening Cohort (SC) and the Internal/Temporal cohort (ITC) were based on the German AD(H)PKD registry. This registry enrolls patients with ADPKD in CKD stages 1–4 since 2015. Information regarding the EC can be found here https://pmc.ncbi.nlm.nih.gov/articles/PMC4042404/<br><br>Serum samples obtained from patients with immunoglobulin A nephropathy (IgAN) who were included into the randomized STOP-IgAN trial were used as controls.
Ethics oversight	Institutional Review Board of the University of Cologne, leading ethics committee at the RWTH Aachen University Hospital (# EK159/07) and local ethics committee at each of the participating centers

Note that full information on the approval of the study protocol must also be provided in the manuscript.

Field-specific reporting

Please select the one below that is the best fit for your research. If you are not sure, read the appropriate sections before making your selection.

[x] Life sciences [ ] Behavioural & social sciences [ ] Ecological, evolutionary & environmental sciences

For a reference copy of the document with all sections, see nature.com/documents/nr-reporting-summary-flat.pdf

Life sciences study design

All studies must disclose on these points even when the disclosure is negative.

Sample size	No formal sample size calculation was performed because this is an exploratory proteomic study aimed at hypothesis generation. Sample size was based on feasibility and precedent, with findings intended to guide future confirmatory studies.
Data exclusions	We excluded MAYO Class 2 patients from our analyses due to unavailability of htTKV. We also excluded samples from patients who are or were on tolvaptan
Replication	We used cross validation approach to mitigate over-fitting and increase the reproducibility. Imputation - In the proteome data, we imputed proteins which have less than 80% missing values. Imputation was done only once by randomly sampling from the 5th percentile of the data.
Randomization	not applicable
Blinding	not applicable

Reporting for specific materials, systems and methods

We require information from authors about some types of materials, experimental systems and methods used in many studies. Here, indicate whether each material, system or method listed is relevant to your study. If you are not sure if a list item applies to your research, read the appropriate section before selecting a response.

Materials & experimental systems

n/a	Involved in the study
☒	☐ Antibodies
☒	☐ Eukaryotic cell lines
☒	☐ Palaeontology and archaeology
☒	☐ Animals and other organisms
☐	☒ Clinical data
☒	☐ Dual use research of concern
☒	☐ Plants

Methods

n/a	Involved in the study
☒	☐ ChIP-seq
☒	☐ Flow cytometry
☒	☐ MRI-based neuroimaging

Clinical data

Policy information about clinical studies
All manuscripts should comply with the ICMJEguidelines for publication of clinical research and a completedCONSORT checklist must be included with all submissions.

Clinical trial registration

Samples measured are part of NCT02497521, DRKS00008910, for EC NCT01616927, for IgA NCT00554502

Study protocol

The study at hand is not a clinical trial but uses biosamples from clinical cohorts (partly derived from clinical trials). However the proteomics study itself is not a clinical trial by nature, i.e. no formal trial protocol or consort checklist applicable.

Data collection

The Screening Cohort (SC) and the Internal/Temporal cohort (ITC) were based on the German AD(H)PKD registry. This registry enrolls patients with ADPKD in CKD stages 1–4 since 2015. Annual clinical and laboratory, imaging parameters, genetics were collected. We analyzed the serum proteome of the Screening Cohort using a semi-automated mass spectrometry pipeline and developed models predicting eGFR slope (214 patients). These models were validated on the Internal/Temporal Cohort (305 patients) and the External Cohort (173 patients).

External Cohort (EC) was from DIPAK cohort and here is the link for data collection:
https://pmc.ncbi.nlm.nih.gov/articles/PMC4042404/

Serum samples obtained from patients with immunoglobulin A nephropathy (IgAN) who were included into the randomized STOP-IgAN trial were used as controls.

Outcomes

not applicable

Plants

Seed stocks

not applicable

Novel plant genotypes

not applicable

Authentication

not applicable
